# Systematic review and meta-analysis of COVID-19 mRNA vaccine effectiveness against hospitalizations in adults

**DOI:** 10.1093/immadv/ltae011

**Published:** 2024-11-27

**Authors:** Bill Kang-Fai Wong, Neil A Mabbott

**Affiliations:** Edinburgh Medical School: Biomedical Sciences, University of Edinburgh, Old Medical School, Teviot Place, Edinburgh EH8 9AG, United Kingdom; The Roslin Institute & Royal (Dick) School of Veterinary Studies, University of Edinburgh, Easter Bush, Midlothian EH25 9RG, United Kingdom

**Keywords:** COVID-19, vaccine efficacy, severe acute respiratory syndrome coronavirus 2, VOC, variant of concern, vaccination

## Abstract

**Background:**

During the coronavirus disease 2019 (COVID-19) pandemic, Pfizer/BioNTech BNT162b2, and Moderna mRNA-1273 vaccines were central to the global pandemic control measures.

**Methods:**

Here, we conducted a systematic review and meta-analysis to evaluate their real-world vaccine effectiveness (VE). Our study focussed on those that reported the efficacy of these vaccines against COVID-19 hospitalization. Hospitalization was chosen as the primary outcome as it directly reflects the ability of the vaccine to prevent severe disease. A literature search was undertaken using Medline and Embase on 25 February 2024. From this, 50 studies out of 18,347 articles were included for further analysis.

**Results:**

High VE against hospitalization was reported for both the BNT162b2 and mRNA-1273 COVID-19 vaccines when used either as a primary vaccination series (2-dose) or following an additional booster dose (3-dose). Meta-analysis indicated that the pooled VE estimates for each of these vaccination protocols ranged from 84% to 86%, suggesting strong protectiveness. Our data also imply that booster doses can restore waning effectiveness, with no significant differences observed in VE between the 2-dose and 3-dose protocols. However, subgroup analysis revealed an association between the presence of the Omicron variant and a drop in VE, indicating that future emerging SARS-CoV-2 virus variants could similarly affect VE.

**Conclusions:**

Our review underscores the importance of ongoing research to ensure vaccine strategies remain effective against evolving variants. Our study also identified the need for expanding data collection to include underrepresented populations.

## Graphical Abstract

**Graphical Abstract:**
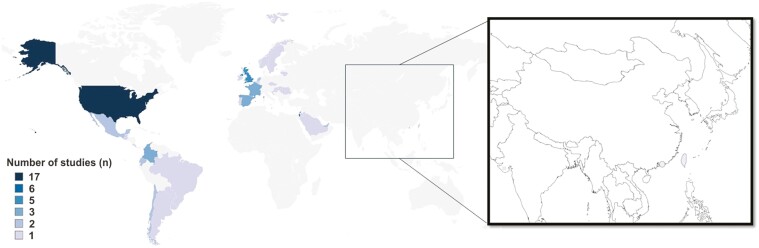

## Introduction

Coronavirus disease 2019 (COVID-19) is caused by infection with severe acute respiratory syndrome coronavirus 2 (SARS-CoV-2), a positive-sense, single-stranded RNA virus of the genus *Betacoronavirus*. The SARS-CoV-2 coronavirus shares a lineage with SARS-CoV-1 and MERS-CoV, both responsible for previous severe respiratory syndromes [1]. The virus’s spike proteins, essential for infection and the primary target of the host immune response, facilitate its high transmissibility [2]. Early in the pandemic, 81% of reported COVID-19 cases exhibited mild symptoms, such as cough and fever, while 14% developed severe symptoms including dyspnoea, with 5% progressing to critical conditions such as respiratory failure and multiple organ dysfunction [3, 4]. The reproductive number (R0) for the original strain of SARS-CoV-2 was estimated at 3.14, significantly higher than that of seasonal influenza (R0 = 1.28) [5, 6]. This high transmissibility contributed to the rapid global spread of the SARS-CoV-2 coronavirus. On 30 January 2020, the World Health Organization declared COVID-19 a public health emergency. As of March 2024, the pandemic had resulted in over 7 million deaths and 774 million cases worldwide [7].

Despite the development and deployment of various COVID-19 vaccines during the pandemic, significant gaps remain in our understanding of their real-world effectiveness. The Pfizer/BioNTech BNT162b2 and Moderna mRNA-1273 COVID-19 vaccines, which were rapidly developed using mRNA technology, demonstrated high efficacy in clinical trials, with reported vaccine efficacy (VE) rates against COVID-19 infection of 95% and 94.1%, respectively [8, 9]. However, translating these efficacy rates into real-world effectiveness is complex. Vaccine effectiveness (VE) in practical settings can be influenced by demographic variations, storage conditions, as well as vaccine uptake levels [10]. In addition, the emergence of SARS-CoV-2 variants of concern (VOCs), such as Alpha, Beta, Gamma, Delta, and Omicron, which possess mutations affecting viral transmissibility and immune escape, further complicate this assessment [11, 12]. Studies have indicated that these variants, particularly Delta and Omicron, reduce VE, underscoring the necessity for continuous evaluation of vaccine performance in response to evolving viral strains [13].

In our study, we systematically reviewed and meta-analysed the real-world effectiveness of the Pfizer/BioNTech BNT162b2 and Moderna mRNA-1273 vaccines against COVID-19 hospitalization. Specifically, the objectives were to: (1) summarize the existing research undertaken on the VE of the BNT162b2 and mRNA-1273 COVID-19 mRNA vaccines against hospitalization; (2) assess the VE of primary series and booster doses throughout the pandemic; and (3) investigate potential differences in VE during the emergence of different VOCs. By focussing on hospitalization as the primary endpoint, our study aimed to provide insights into each vaccine’s effectiveness in preventing severe disease, which is critical for public health planning and resource allocation. This analysis will aid our understanding of mRNA COVID-19 vaccine performance in an evolving pandemic landscape.

## Materials and methods

### Search strategy

A literature search was conducted on 25 February 2024 using two online databases: Embase (https://www.embase.com/) and Medline (https://www.nlm.nih.gov/medline/). The concept words and search terms used in the search are presented in Table 1. The same search strategy was applied to both databases. The search strategy was designed to cover all the literature reporting the effectiveness or efficacy of any vaccine format against COVID-19 independent of vaccination protocols, vaccine design or effectiveness endpoints.

**Table 1.** A table showing the search strategy designed for the literature search. The concept words and search terms used in the literature search were shown.

Concept words	Search terms
COVID-19	‘SARS-CoV-2’ OR ‘COVID-19’ OR ‘COVID’
AND	
Vaccine	‘vaccine’ OR ‘vaccination’ OR ‘booster*’ OR ‘third dose’ OR ‘fourth dose’ OR ‘fifth dose’
AND	
Effectiveness	‘effectiveness’ OR ‘efficacy’

### Study eligibility criteria

The eligibility criteria were designed in alignment with the PICOS framework, which outlines the scope of a review based on population, intervention, comparison, outcome, and study type [14]. The Preferred Reporting Items for Systematic Reviews and Meta-Analyses (PRISMA) checklist is provided in the Supplementary information. A comprehensive list of the inclusion and exclusion criteria used in this study is provided in Table 2. Studies were limited to those that reported VE for the Pfizer/BioNTech BNT162b2 and Moderna mRNA-1273 COVID-19 vaccines. Hospitalization was selected as the sole VE endpoint in this review as it directly reflects the ability of the vaccine to prevent severe disease. Thus, studies not reporting VE for the two vaccines of interest or reporting VE for outcomes other than hospitalization were excluded (Table 2). Studies reporting VE in an at-risk population subgroup such as health care workers or elderly care home residents were also excluded, as these subgroups may not provide a representative assessment of VE in the broader community [15, 16]. The review included studies reporting absolute VE and excluded those reporting relative VE. Absolute VE was defined as the reduction in COVID-19 hospitalization in the vaccinated group relative to an unvaccinated group. Relative VE, in contrast, compares the reduction of disease between different vaccination protocols [17].

**Table 2.** Inclusion and exclusion criteria were designed based on the PICOS framework. Only studies that met all the inclusion criteria were included in the analysis.

PICOS	Inclusion criteria	Exclusion criteria
Population	Studies reporting findings on adults (>18)	Animal studies; Studies focussing on demographics with comorbidities; Studies focussing on at-risk populations, for example, healthcare workers, elder care home residents; Studies reporting findings on children or adolescents (<18) only; Studies reporting findings on the elderly (>65) only
Intervention	Any dosage of monovalent Pfizer-BioNTech BNT162b2 vaccine; Any dosage of monovalent Moderna mRNA-1273 vaccine	Any other COVID-19 vaccines; Any bivalent COVID-19 vaccine
Comparison	Comparing VE between vaccinated group and unvaccinated population group	Comparing VE between different vaccination protocols, for example, comparing VE of 2-dose BNT162b2 against 3-dose BNT162b2
Outcome	Absolute VE (%) against COVID-19 hospitalization	Studies reporting VE on outcomes other than hospitalization, for example, infection, severe disease, death, and psychological impact; Studies with incomplete data; Studies reporting VE in effect sizes (e.g. risk ratio, odds ratio, or hazard ratio) instead of percentages
Study type	Cohort studies; Case-control studies; Test-negative studies; Registry-based studies	Studies not reporting on primary data, for example, reviews, systematic reviews; Case reports or case series; Opinion pieces; Correspondence; Studies that were not peer-reviewed; Studies not written in English

### The Covidence App for duplicate removal, title, and abstract screening

The literature search results were uploaded to the Covidence App (https://www.covidence.org/), a web-based software platform for the automatic identification and removal of duplicates. Additional duplicates identified in the screening process were removed manually. After removing duplicates, the titles and abstracts were screened in the remaining unique articles based on the eligibility criteria presented in Table 2.

### Data extraction

Data extraction from the full texts of the eligible studies was conducted and archived using Microsoft Excel (Version 2403). Data extracted from eligible studies included the study characteristics, VE% against COVID-19 hospitalization and the vaccination protocols. The entire review process, including study identification, screening, and data extraction, was performed by BKFW.

## Statistical analysis

Data processing and preliminary analyses were primarily performed in Microsoft Excel. Advanced statistical analyses and data visualization were undertaken using RStudio (version 4.2.2). The forest plots were generated using the R packages ‘ggplot2’, ‘dplyr’, and ‘patchwork’. Given the high heterogeneity observed among the included studies, a meta-analysis was conducted using a random effects model using the metafor package. This approach accounts for variability across studies and is suitable when there is significant heterogeneity [18]. The *I*^2^ statistic, which measures the proportion of total variability due to heterogeneity, was calculated to evaluate the consistency of results among the studies [19].

## Results

### Characteristics of the included studies

**Figure 1.**
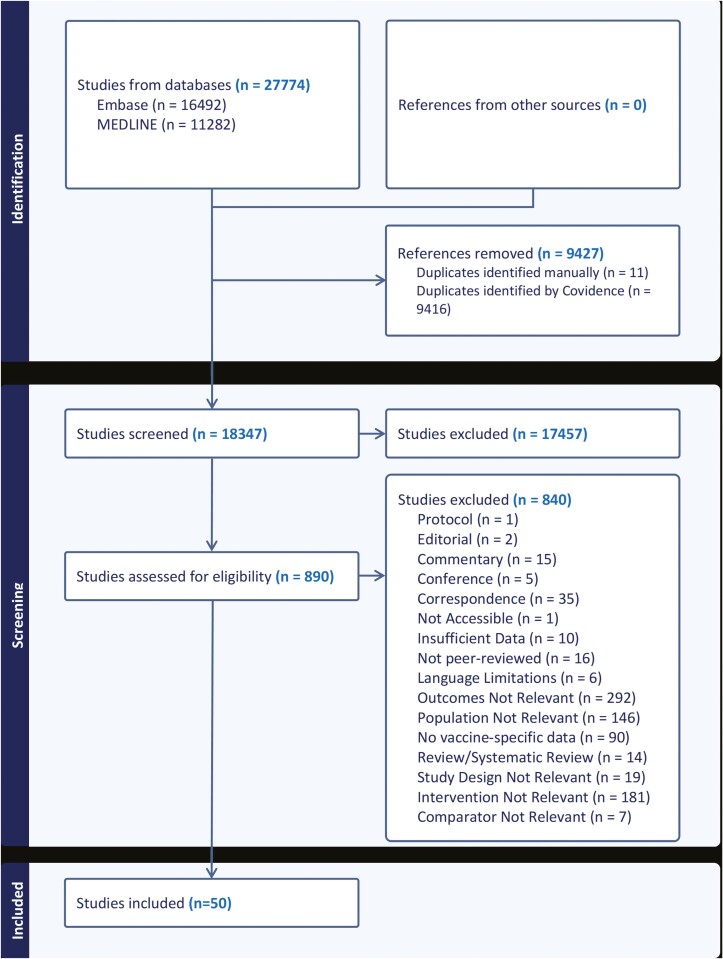
PRISMA flow diagram depicting the number of studies at each stage of the review process. The flowchart was generated using the Covidence App.

The literature search results, structured according to the Preferred Reporting Items for Systematic Reviews and Meta-Analyses (PRISMA) guidelines (Supplementary information) are presented in Fig. 1 [20]. Initially, 16,492 records were identified from Embase and 11,282 records from Medline. Upon removal of 9,427 duplicates, of which 11 records were identified manually, 18,347 unique studies underwent title and abstract screening. Subsequently, 890 studies were eligible for full-text screening, during which 839 studies were excluded as they did not match the inclusion criteria for the study, such as VE measures other than hospitalization (Fig. 1). Ultimately, 50 studies met the inclusion criteria and were included in this review. The details of each included study are provided in Supplementary Table 1.

Of the 50 studies that met the eligibility criteria, the majority (30 out of 50) were conducted between the years 2021 and 2022. More than half of the studies reported multiple VE readings, with a total of 92 records extracted. Approximately three-quarters (37/50) of the studies had sample sizes ranging from 10,000 to 10 million participants. The term “sample size” in Table 3 refers to the total number of participants or data included in each study. This encompasses the initial cohort size for cohort studies, and the combined number of cases and controls in case-control studies but does not indicate the number of subjects per vaccination regimen. Regarding the predominant coronavirus VOCs during the study period, the majority of the studies reported VE during the Delta-predominant period (*n* = 22), followed by Omicron (*n* = 14) and Alpha (*n* = 13). Only nine studies made no mention of the circulating coronavirus variants at the time the study was conducted.

**Table 3.** Summary of characteristics of the included studies. The study period, vaccination protocol, sample size, and prevalent variant of concern of the included studies were reported.

Study characteristics	Number of studies
Study period	
2020–2021	16
2021 only	19
2021–2022	11
2022 only	4
Vaccination protocol	
2-dose BNT162b2	44
2-dose mRNA-1273	26
3-dose BNT162b2	15
3-dose mRNA-1273	7
Sample size	
Under 10 thousand	10
10 thousand—1 million	19
1–10 million	18
Over 10 million	3
Prevalent variant of concern (VOC)	
Alpha	13
Gamma	2
Delta	22
Omicron	14
Did not specified	9

### Geographical distribution of the included studies

**Figure 2.**
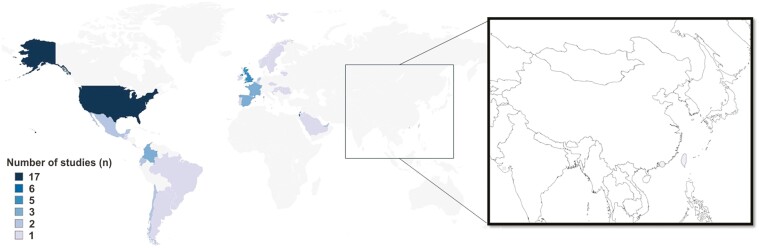
Map of the world showing the geological distribution of the included studies. The colour scale depicts the number of included studies from each region. No data were collected from the countries within the grey-shaded regions.

The largest proportion of studies included in the current review reported VE data against hospitalization from the United States, representing 17 out of the 50 included studies (Fig. 2). Six studies reported data from Israel, with five studies were included from the United Kingdom. The geographical distribution of the included studies is presented in Fig. 2.

Europe and South America also contributed to the body of research analysed, though to a lesser extent, indicating a more dispersed research presence across these continents. Notably, no studies were included from Africa, highlighting an important data gap for this geographic region. In Asia, only data from two studies from Hong Kong and one study from Taiwan contributed to this study, indicating limited representation of VE data from regions outside of Europe, North America, and South America.

### High vaccine effectiveness for the BNT162b2 and mRNA-1273 vaccines after a primary series and one booster dose

A total of 92 records of VE were extracted from the 50 studies. Among these, 44 records included data for VE after a primary series (2-dose) protocol using the BNT162b2 mRNA COVID-19 vaccine, and 26 records described VE after the primary series protocol using the mRNA-1273 COVID-19 vaccine. In comparison, there were fewer records of VE after one booster dose (3-dose) for either of the mRNA vaccines (BNT162b2, 15 records; mRNA-1273, 7 records).

**Figure 3.**
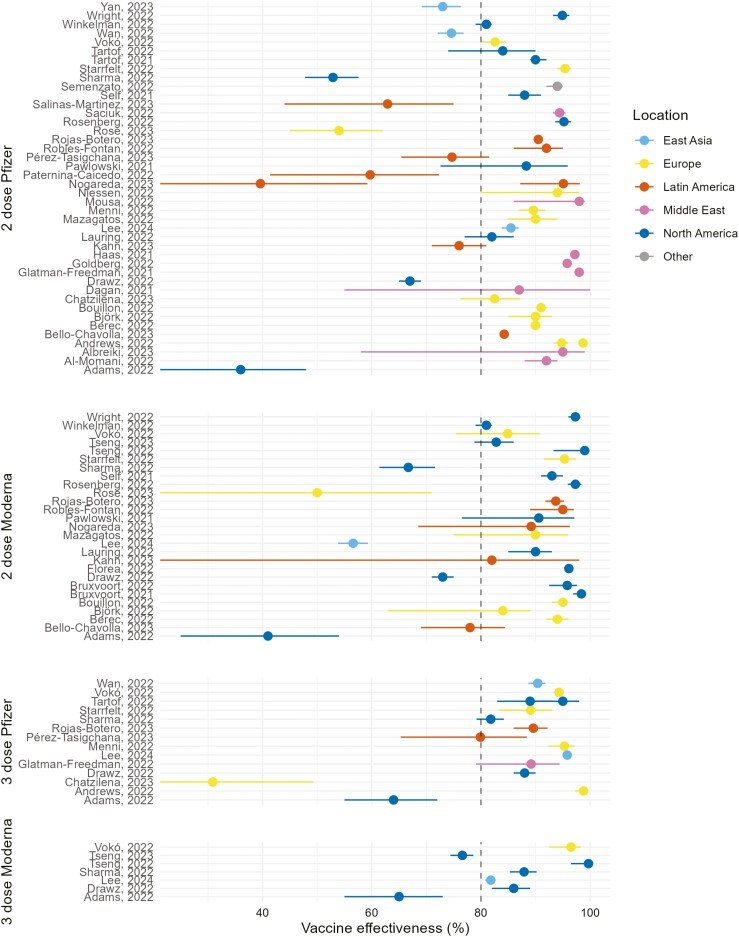
Forest plot showing reported VE% in each of the eligible studies. The plot shows the VE% against hospitalization for the BNT162b2 and mRNA-1273 COVID-19 vaccines after both a primary series (2-dose) and booster (3-dose) protocol. The vertical dotted reference line denotes 80% VE against hospitalization. The plot is colour-coded to represent the geographical region of each study.

The VE records were extracted from the included studies and presented in a forest plot grouped by each of the four vaccination protocols (vaccine type, no. doses; Fig. 3). Despite some heterogeneity between the studies, irrespective of the vaccine or vaccination protocol used, the reported VE data for most studies was positioned to the right-hand side of (above) the 80% VE reference line (Fig. 3). This suggested that both the BNT162b2 and mRNA-1273 COVID-19 vaccines provided strong VE against COVID-19 hospitalization.

### Pooled vaccine effectiveness estimates for each vaccination protocol

To better understand the overall VE of the four vaccination protocols, a meta-analysis was undertaken to calculate the pooled estimates of VE. By using pooled estimates, this enabled VE extracted from studies conducted throughout the pandemic to be considered. A random effects model was used to enable the heterogeneity observed in Fig. 3 to be taken into account. This meta-analysis suggested the pooled VE estimates against hospitalization across the four vaccination protocols ranged from 84.34% to 86.71% (Table 4), indicating high overall VE for both the BNT162b2 and mRNA-1273 COVID-19 vaccines.

**Table 4.** Pooled estimates of VE% of the four regimens. The pooled estimates were provided along with the *I*^2^ statistics, which measure the degree of heterogeneity among the study results.

Vaccination protocol	Pooled VE estimates (%)	*I* ^2^	*P* value
2-dose BNT162b2	84.34 (80.27–88.40)	99.81%	<.0001
2-dose mRNA-1273	86.02 (80.76–91.32)	99.44%	<.0001
3 dose BNT162b2	86.71 (80.56–92.86)	99.35%	<.0001
3 dose mRNA-1273	85.17 (76.78–93.55)	99.07%	<.0001

In addition, there were no significant differences in the pooled VE estimates between the primary series and one booster dose for both BNT162b2 and mRNA-1273 COVID-19 vaccines, as their confidence intervals largely overlap (Table 4). This implies that for each of the COVID-19 mRNA vaccines analysed, one homologous booster dose was sufficient to restore the VE of the primary series.

No significant differences in VE were also identified between the primary series and one booster dose protocol for either vaccine. This suggests that the BNT162b2 and mRNA-1273 COVID-19 vaccines were similarly effective throughout the pandemic, with little if any indication of either mRNA vaccine being more effective than the other.

However, the meta-analysis also revealed substantial heterogeneity among the studies, with *I*^2^ values exceeding 99% and high significance for each of the VE estimates (Table 4). This high degree of heterogeneity suggests that much of the variability among these studies was due to intrinsic differences in study designs rather than sampling error. Thus, the pooled VE estimates should be interpreted with caution.

### Reduced vaccine effectiveness in presence of the Omicron coronavirus variant

**Figure 4.**
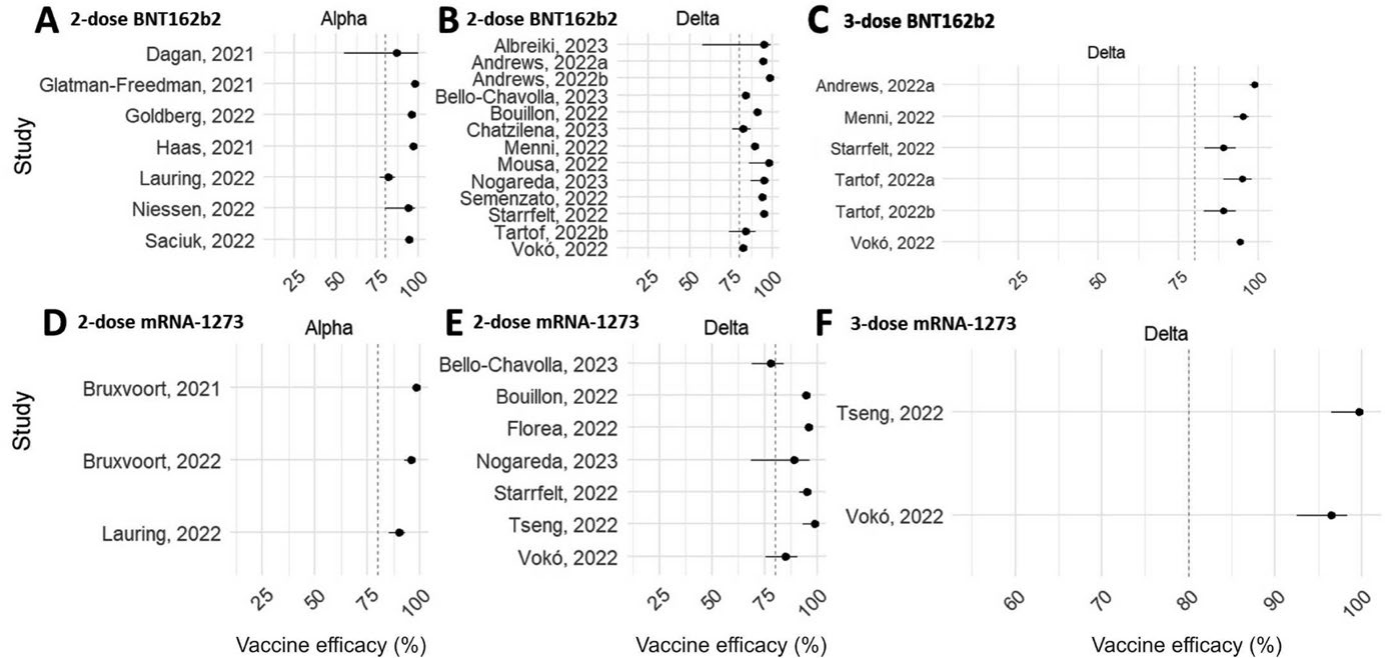
Forest plots showing VE of both BNT162b2 and mRNA-1273 COVID-19, for both the primary (2 dose) and one booster (3 dose) series against the Alpha and Delta coronavirus VOC. (A–C), BNT162b2. (D–F), mRNA-1273. The vertical dotted line represents 80% VE for reference.

**Figure 5.**
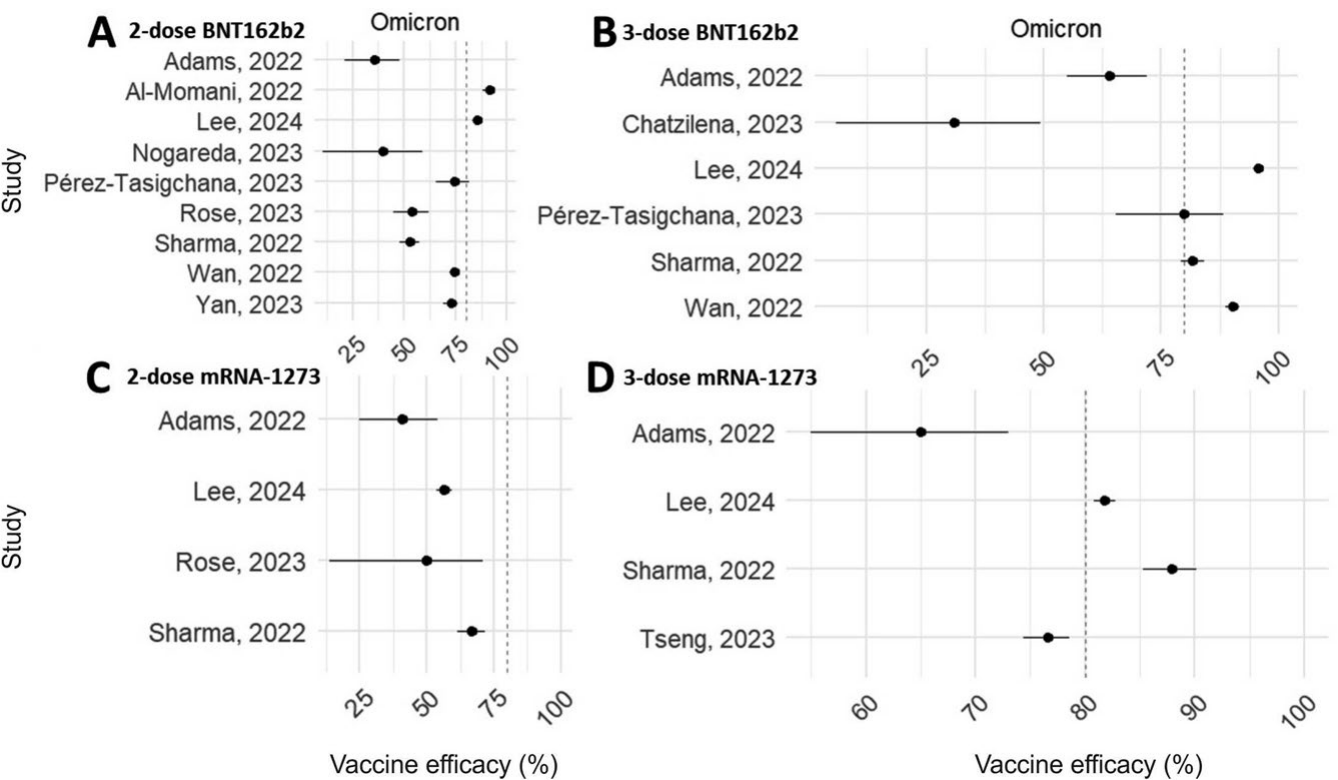
Forest plots showing VE of both BNT162b2 and mRNA-1273 COVID-19, for both the primary (2 dose) and one booster (3 dose) series against the Omicron coronavirus VOC. (A and B), BNT162b2. (C and D), mRNA-1273. The vertical dotted line represents 80% VE for reference.

To address the significant heterogeneity in VE, a subgroup analysis of VE was undertaken based on the predominant coronavirus VOC that was prevalent at the time the studies were undertaken. As described in Table 3, most included studies reported VE under the prevalence of the Alpha, Delta, and Omicron coronavirus variants. The VE for primary and one booster dose series for both BNT162b2 and mRNA-1273 COVID-19 vaccines grouped by the VOC are shown in Figs 4 and 5. During the prevalence of Alpha and Delta coronavirus variants, the VE for each vaccine typically exceeded 80%, indicating robust protection against hospitalization after infection with these variants (Fig. 4). However, a notable reduction in VE was observed with the emergence of the Omicron coronavirus variant. Our analysis showed that VE was typically lower during periods when Omicron was prevalent, with most estimates falling below the 80% reference line regardless of the vaccination protocol (Fig. 5).

## Discussion

Our systematic review summarized the results of 50 distinct studies published between 2021 and 2024, which reported VE against COVID-19 hospitalization for patients immunized with a primary (2-dose) or one booster (3-dose) protocol using either the BNT162b2 (Pfizer/BioNTech) or mRNA-1273 (Moderna) mRNA COVID-19 vaccines. Across the four vaccination protocols, the majority of the included studies reported VE above 80%, suggesting that these mRNA vaccines were associated with robust protection against COVID-19 hospitalization. Meta-analysis of the included studies reported consistent results, with the pooled estimates of VE of the four vaccination protocols ranging between 84.34% and 86.71%. However, subgroup analysis of VE by the prevalence of VOC revealed that the predominance of the Omicron variant in the circulating strains was associated with a decrease in VE.

Our study aimed to assess the real-world VE of the BNT162b2 and mRNA-1273 mRNA COVID-19 vaccines. We selected COVID-19 hospitalization as the sole VE endpoint for multiple reasons. Firstly, it reflects vaccine protectiveness against severe disease directly, compared to infection where mild disease is also recorded [21]. Secondly, the selection of hospitalization allows for higher consistency across different studies as definitions for COVID-19 hospitalization are less likely to be influenced by variations in testing methods, providing a more objective and reliable basis for comparing VE across independent studies [22]. In addition, hospitalization has a direct impact on healthcare systems, influencing hospital capacity, resource allocation, and overall healthcare burden [23]. We included studies that reported VE against COVID-19 hospitalization in percentages, whereas those that reported VE in effect sizes such as risk ratios or odds ratios were excluded. Including VE in percentages only, ensured direct comparability between the included studies and simplified data interpretation.

Comparison of our pooled VE estimates against hospitalization for a primary series of the BNT162b2 and mRNA-1273 mRNA COVID-19 vaccines with those for other vaccine formats shows they have a similar or higher level of VE. For example, the VE against hospitalization for a primary series of the BBIBP-CorV (Sinopharm) inactivated COVID-19 vaccine was estimated to be 79.6% (77.7–81.3) [24], the inactivated CoronaVac inactivated vaccine has a VE of 87.5% (86.7–88.2%) [25] and the Ad26.COV2.S viral vector COVID-19 vaccine has a VE of 72% (64–77%) [26].

Our subgroup analysis revealed an observable drop in VE during the period when Omicron was the predominant VOC. This association between the presence of the Omicron coronavirus variant and a reduction in VE is likely due to the presence of many inherent mutations within the Omicron strain that confer immune escape. Relative to the ancestral strain of SARS-CoV-2, the Omicron strain carries 60 mutations, 32 of which are located within the spike protein region [27]. As the mRNA vaccines were designed to deliver genetic information to produce coronavirus spike protein-specific antibodies, mutations in this region are likely to reduce the binding specificity of the vaccine-induced antibodies, leading to decreased neutralization and lower VE. A study by Cele *et al.* [28] provided supporting evidence by testing the ability of plasma from 19 individuals who had completed the primary series of BNT162b2 to neutralize the Omicron variant. They found a 22-fold reduction in vaccine-elicited neutralization against the Omicron variant. This decline in VE has also been observed in VE studies targeting symptomatic infection. Florentino *et al.* [29] reported 14.0% and 10.2% lower VE of a 2-dose course of the BNT162b2 vaccine against symptomatic infection in adolescents in Brazil and Scotland, respectively, when compared to the periods when the Delta and Omicron variants were predominant.

To address the reduced VE associated with the emergence of the Omicron variant, bivalent mRNA vaccines containing the mRNA encoding the spike proteins of both the ancestral Wuhan-1 strain and the Omicron strain were developed [30]. Tartof *et al*. [31] reported a 50% (23%–68%) higher VE against critical illness and a 39% (28%–49%) higher VE against hospital admission for the BNT162b2 BA.4/5 bivalent vaccine compared to 2 doses of BNT162b2 or mRNA-1273. The subsequent emergence of sub-lineages of the Omicron variant that also exhibit lower susceptibility to the monovalent mRNA COVID-19 vaccines underscores the need to regularly update the COVID-19 vaccines to maintain protectiveness against emerging and circulating coronavirus VOCs [32].

This review provides indirect evidence for the effect of a booster dose in rescuing the waning VE of a primary immunization series against hospitalization. Although the included studies reported different lengths of follow-up periods, the pooled VE estimates for the 2-dose and 3-dose protocols for each of the mRNA COVID-19 vaccines analysed overlapped. This suggested that the VE conferred by these two immunization protocols were similar. This alignment in VE between the 2-dose and 3-dose protocols implies that administering a subsequent booster dose can effectively restore VE. Indeed, among the included studies that provided data for both a primary series and one booster dose of BNT162b2 or mRNA-1273, the VE of the booster dose was always higher than primary series [33, 34]. Bar-on *et al.* [35] has similarly reported that compared with the primary series of immunization with BNT162b2 a booster dose resulted in a lower rate of COVID-19 infection by a factor of 11.3.

Notably, our analysis identified outliers in VE, specifically the studies by Adams *et al.* [36] and Chatzilena *et al.* [37], which reported significantly lower VE for the 3-dose regimens of BNT162b2 and mRNA-1273, respectively, compared to other studies. These discrepancies may be attributed to the majority of the sample populations in these studies receiving the ancestral strain vaccine rather than the updated bivalent vaccine. In contrast, the studies reporting higher VE for the 3-dose regimens may include a mix of both ancestral and bivalent vaccines, potentially leading to enhanced effectiveness against variants. Although this review provides insights into the VE of two mRNA vaccines against COVID-19 hospitalization, we acknowledge some potential limitations. While the current study did not impose any geographical constraints in the selection of studies, we nonetheless identified an uneven distribution of the studies, with the majority reporting data from the Americas and Europe, with limited representation from Asia, and no studies from Africa. Some COVID-19 mRNA VE studies have been published in Singapore [38] and Japan [39]. However, these were excluded during the screening process since they did not report VE against COVID-19 hospitalization or study effects of the bivalent mRNA vaccines. Whether the VE estimates reported in this review are applicable to distinct populations across the world is uncertain, as many factors unique to these regions may have an important influence. For example, helminth parasite infections are prevalent in people in low- and middle-income countries [40] and the immune responses to them can affect the efficacy of some vaccines [41, 42]. It is, therefore, essential that studies are also conducted in low- and middle-income countries to enable the impact of factors such as co-infection with other pathogens have on VE [43].

In addition, this review did not account for the impact of follow-up duration on VE, as the reported follow-up periods varied significantly among the included studies. Some studies reported follow-up durations of over 7 days, with others reporting durations extending beyond 210 days. This variability in reporting indicates a lack of standardization in how follow-up durations are defined and documented across different studies. Such inconsistencies can complicate the interpretation of VE results, as the timing of follow-up can influence the observed effectiveness of vaccines. Short follow-up periods may not capture the full extent of waning immunity, while longer follow-up durations provide insights into the durability of the immune response over time. Furthermore, very few studies specified follow-up durations for each mRNA vaccine, which limits our ability to assess how differences in vaccine type and administration timing may affect VE. This gap in data underscores the need for future research to adopt standardized protocols for reporting follow-up durations, enabling more accurate comparisons and a clearer understanding of the long-term effectiveness of mRNA vaccines against COVID-19.

Stringent transportation and storage requirements impact the distribution of the COVID-19 mRNA vaccines since these require cold-chain storage, in which the Pfizer vaccine has to be kept at −60°C [44]. This greatly limits mRNA vaccine access in regions that lack access to reliable storage and transportation facilities. As such, among the different brands of vaccine supplied to Africa under the COVID-19 Vaccines Global Access (COVAX) initiative, the Oxford/AstraZeneca vaccine was the most widely distributed since it can be safely stored at 2–8°C and does not require the same logistical requirements as the mRNA vaccines [45]. These conditions may contribute to the lack of COVID-19 mRNA VE studies from African and South-East Asian regions.

## Conclusion

In conclusion, this systematic review of 50 independent studies suggests that the Pfizer/BioNTech BNT162b2 and Moderna mRNA-1273 mRNA vaccines confer robust protection against COVID-19 hospitalization after a primary or booster immunization series. The study also highlighted how different VOCs such as the Omicron variant could impact on VE, underscoring the importance of ongoing research to ensure vaccine strategies remain effective against evolving variants. Our study also identified the need for expanding data collection to include underrepresented populations.

## Supplementary Material

ltae011_suppl_Supplementary_Data

## Data Availability

The data underlying this article are available in the article and in its online supplementary material.

## Abbreviations

COVID-19: coronavirus disease 2019
PRISMA: preferred reporting items for systematic reviews and meta-analyses
SARS-CoV-2: severe acute respiratory syndrome coronavirus 2
VE: vaccine efficacy
VOC: variant of concern
WHO: World Health Organization

## Clinical trial registration

This review was not registered and the protocol was not prepared.

## References

[1] De Wit E , Van Doremalen N, Falzarano D et al SARS and MERS: recent insights into emerging coronaviruses. Nat Rev Microbiol 2016; 14(8):523–34. https://doi.org/10.1038/nrmicro.2016.81 PMC7097822 27344959

[2] Hoffmann M , Kleine-Weber H, Schroeder S et al SARS-CoV-2 cell entry depends on ACE2 and TMPRSS2 and is blocked by a clinically proven protease inhibitor. Cell 2020; 181(2):271–280.e8.e8. https://doi.org/10.1016/j.cell.2020.02.052 32142651 PMC7102627

[3] Wu Z , Mcgoogan JM. Characteristics of and important lessons from the Coronavirus Disease 2019 (COVID-19) outbreak in China: summary of a report of 72 314 cases from the Chinese Center for Disease Control and Prevention. JAMA 2020; 323(13):1239–42. https://doi.org/10.1001/jama.2020.2648 32091533

[4] Lamers MM , Haagmans BL. SARS-CoV-2 pathogenesis. Nat Rev Microbiol 2022; 20(5):270–84. https://doi.org/10.1038/s41579-022-00713-0 35354968

[5] Billah MA , Miah MM, Khan MN. Reproductive number of coronavirus: a systematic review and meta-analysis based on global level evidence. PLoS One 2020; 15(11):e0242128. https://doi.org/10.1371/journal.pone.0242128 33175914 PMC7657547

[6] Biggerstaff M , Cauchemez S, Reed C et al Estimates of the reproduction number for seasonal, pandemic, and zoonotic influenza: a systematic review of the literature. BMC Infect Dis 2014; 14:480. https://doi.org/10.1186/1471-2334-14-480 25186370 PMC4169819

[7] WHO. 2024. *WHO COVID-19 Dashboard*. https://data.who.int/dashboards/covid19/cases?n=c (10 February 2024, date last accessed).

[8] Polack FP , Thomas SJ, Kitchin N et al; C4591001 Clinical Trial Group. Safety and efficacy of the BNT162b2 mRNA Covid-19 vaccine. N Engl J Med 2020; 383(27):2603–15. https://doi.org/10.1056/NEJMoa2034577 33301246 PMC7745181

[9] Baden LR , El Sahly HM, Essink B et al; COVE Study Group. Efficacy and safety of the mRNA-1273 SARS-CoV-2 vaccine. N Engl J Med 2020; 384(5):403–16. https://doi.org/10.1056/NEJMoa2035389 33378609 PMC7787219

[10] Weinberg GA , Szilagyi PG. Vaccine epidemiology: efficacy, effectiveness, and the translational research roadmap. J Infect Dis 2010; 201(11):1607–10. https://doi.org/10.1086/652404 20402594

[11] Harvey WT , Carabelli AM, Jackson B et al SARS-CoV-2 variants, spike mutations and immune escape. Nat Rev Microbiol 2021; 19(7):409–24. https://doi.org/10.1038/s41579-021-00573-0 34075212 PMC8167834

[12] Abbasian MH , Mahmanzar M, Rahimian K et al Global landscape of SARS-CoV-2 mutations and conserved regions. J Transl Med 2023; 21(1):152. https://doi.org/10.1186/s12967-023-03996-w 36841805 PMC9958328

[13] Paul P , El-Naas A, Hamad O et al Effectiveness of the pre-Omicron COVID-19 vaccines against Omicron in reducing infection, hospitalization, severity, and mortality compared to Delta and other variants: a systematic review. Hum Vaccin Immunother 2023; 19(1):2167410. https://doi.org/10.1080/21645515.2023.2167410 36915960 PMC10054360

[14] Methley AM , Campbell S, Chew-Graham C et al PICO, PICOS and SPIDER: a comparison study of specificity and sensitivity in three search tools for qualitative systematic reviews. BMC Health Serv Res 2014; 14:579. https://doi.org/10.1186/s12913-014-0579-0 25413154 PMC4310146

[15] Gaio V , Santos AJ, Amaral P et al COVID-19 vaccine effectiveness among healthcare workers: a hospital-based cohort study. BMJ Open 2023; 13(5):e068996. https://doi.org/10.1136/bmjopen-2022-068996 PMC10163328 37130692

[16] Zhang Y , Wang Y, Ning G et al Protecting older people: a high priority during the COVID-19 pandemic. Lancet (London, England) 2022; 400(10354):729–30. https://doi.org/10.1016/S0140-6736(22)01530-6 PMC9436364 36058217

[17] Lewis NM , Murray N, Adams K et al a. Absolute and relative vaccine effectiveness of primary and booster series of COVID-19 vaccines (mRNA and Adenovirus Vector) against COVID-19 hospitalizations in the United States, December 2021–April 2022. Open Forum Infectious Diseases 2022; 10(1):ofac698. https://doi.org/10.1093/ofid/ofac698 36695662 PMC9868348

[18] Riley RD , Higgins JPT, Deeks JJ. Interpretation of random effects meta-analyses. BMJ 2011; 342:d549–d549. https://doi.org/10.1136/bmj.d549 21310794

[19] Higgins JPT , Thompson SG. Quantifying heterogeneity in a meta-analysis. Stat Med 2002; 21(11):1539–58. https://doi.org/10.1002/sim.1186 12111919

[20] Page MJ , Mckenzie JE, Bossuyt PM et al The PRISMA 2020 statement: an updated guideline for reporting systematic reviews. BMJ 2021; 372:n71. https://doi.org/10.1136/bmj.n71 33782057 PMC8005924

[21] Mehrotra DV , Janes HE, Fleming TR et al Clinical endpoints for evaluating efficacy in COVID-19 vaccine trials. Ann Intern Med 2021; 174(2):221–8. https://doi.org/10.7326/M20-6169 33090877 PMC7596738

[22] Filchakova O , Dossym D, Ilyas A et al Review of COVID-19 testing and diagnostic methods. Talanta 2022; 244:123409. https://doi.org/10.1016/j.talanta.2022.123409 35390680 PMC8970625

[23] Garcia-Carretero R , Vazquez-Gomez O, Gil-Prieto R et al Hospitalization burden and epidemiology of the COVID-19 pandemic in Spain (2020–2021). BMC Infect Dis 2023; 23(1):476. https://doi.org/10.1186/s12879-023-08454-y 37464303 PMC10353154

[24] Al Kaabi N , Oulhaj A, Ganesan S et al Effectiveness of BBIBP-CorV vaccine against severe outcomes of COVID-19 in Abu Dhabi, United Arab Emirates. Nat Commun 2022; 13(1):3215. https://doi.org/10.1038/s41467-022-30835-1 35680857 PMC9184465

[25] Jara A , Undurraga EA, González C et al Effectiveness of an inactivated SARS-CoV-2 vaccine in Chile. N Engl J Med 2021; 385(10):875–84. https://doi.org/10.1056/NEJMoa2107715 34233097 PMC8279092

[26] Lewis NM , Self WH, Gaglani M et al; IVY Network Collaborators. Effectiveness of the Ad26.COV2.S (Johnson & Johnson) Coronavirus Disease 2019 (COVID-19) vaccine for preventing COVID-19 hospitalizations and progression to high disease severity in the United States. Clin Infect Dis2022b; 75(Suppl 2):S159–66. https://doi.org/10.1093/cid/ciac439 35675695 PMC9214149

[27] Tarcsai KR , Corolciuc O, Tordai A et al SARS-CoV-2 infection in HIV-infected patients: potential role in the high mutational load of the Omicron variant emerging in South Africa. Geroscience 2022; 44(5):2337–45. https://doi.org/10.1007/s11357-022-00603-6 35739343 PMC9225796

[28] Cele S , Jackson L, Khoury DS et al Omicron extensively but incompletely escapes Pfizer BNT162b2 neutralization. Nature 2022; 602(7898):654–6. https://doi.org/10.1038/s41586-021-04387-1 35016196 PMC8866126

[29] Florentino PTV , Millington T, Cerqueira-Silva T et al Vaccine effectiveness of two-dose BNT162b2 against symptomatic and severe COVID-19 among adolescents in Brazil and Scotland over time: a test-negative case-control study. Lancet Infect Dis 2022; 22(11):1577–86. https://doi.org/10.1016/S1473-3099(22)00451-0 35952702 PMC9359673

[30] Chalkias S , Harper C, Vrbicky K et al A bivalent omicron-containing booster vaccine against Covid-19. N Engl J Med 2022; 387(14):1279–91. https://doi.org/10.1056/NEJMoa2208343 36112399 PMC9511634

[31] Tartof SY , Slezak JM, Puzniak L et al Effectiveness of BNT162b2 BA.4/5 bivalent mRNA vaccine against a range of COVID-19 outcomes in a large health system in the USA: a test-negative case-control study. Lancet Respir Med 2023; 11(12):1089–100. https://doi.org/10.1016/S2213-2600(23)00306-5 37898148

[32] Link-Gelles R , Levy ME, Natarajan K et al Estimation of COVID-19 mRNA vaccine effectiveness and COVID-19 illness and severity by vaccination status during omicron BA.4 and BA.5 sublineage periods. JAMA Network Open 2023; 6(3):e232598–e232598. https://doi.org/10.1001/jamanetworkopen.2023.2598 36920396 PMC10018321

[33] Andrews N , Stowe J, Kirsebom F et al Effectiveness of COVID-19 booster vaccines against COVID-19-related symptoms, hospitalization and death in England. Nat Med 2022; 28(4):831–7. https://doi.org/10.1038/s41591-022-01699-1 35045566 PMC9018410

[34] Sharma A , Oda G, Holodniy M. Effectiveness of messenger RNA-based vaccines during the emergence of the severe acute respiratory syndrome coronavirus 2 omicron variant. Clin Infect Dis 2022; 75(12):2186–92. https://doi.org/10.1093/cid/ciac325 35475889 PMC9129111

[35] Bar-On YM , Goldberg Y, Mandel M et al Protection of BNT162b2 vaccine booster against COVID-19 in Israel. N Engl J Med 2021; 385(15):1393–400. https://doi.org/10.1056/NEJMoa2114255 34525275 PMC8461568

[36] Adams K , Rhoads JP, Surie D et al; Influenza and other Viruses in the AcutelY ill (IVY) Network. Vaccine effectiveness of primary series and booster doses against COVID-19 associated hospital admissions in the United States: living test negative design study. BMJ (Clinical Research ed.) 2022; 379:e072065. https://doi.org/10.1136/bmj-2022-072065 PMC9551237 36220174

[37] Chatzilena A , Hyams C, Challen R et al; Avon CAP Research Group. Effectiveness of BNT162b2 COVID-19 vaccination in prevention of hospitalisations and severe disease in adults with SARS-CoV-2 Delta (B.1.617.2) and Omicron (B.1.1.529) variant between June 2021 and July 2022: a prospective test negative case-control study. Lancet Regulatory Health Europe 2022; 25:100552. https://doi.org/10.1016/j.lanepe.2022.100552 PMC9728025 36506791

[38] Ng OT , Marimuthu K, Lim N et al Analysis of COVID-19 incidence and severity among adults vaccinated with 2-dose mRNA COVID-19 or inactivated SARS-CoV-2 vaccines with and without boosters in Singapore. JAMA Netw Open 2022; 5(8):e2228900. https://doi.org/10.1001/jamanetworkopen.2022.28900 36018588 PMC9419014

[39] Tamada Y , Takeuchi K, Kusama T et al Bivalent mRNA vaccine effectiveness against COVID-19 among older adults in Japan: a test-negative study from the VENUS study. BMC Infect Dis 2024; 24(1):135. https://doi.org/10.1186/s12879-024-09035-3 38287337 PMC10823731

[40] Hotez PJ , Brindley PJ, Bethony JM et al Helminth infections: the great neglected tropical diseases. J Clin Invest 2008; 118(4):1311–21. https://doi.org/10.1172/JCI34261 18382743 PMC2276811

[41] Mabbott NA. The influence of parasite infections on host immunity to co-infection with other pathogens. Front Immunol 2018; 9:2579. https://doi.org/10.3389/fimmu.2018.02579 30467504 PMC6237250

[42] Wait LF , Dobson AP, Graham AL. Do parasite infections interfere with immunisation? A review and meta-analysis. Vaccine 2020; 38(35):5582–90. https://doi.org/10.1016/j.vaccine.2020.06.064 32616328

[43] Egwang TG , Owalla TJ, Kemigisha M. COVID-19 vaccine trials must include helminth-infected cohorts. Nat Immunol 2022; 23(2):148. https://doi.org/10.1038/s41590-021-01116-8 35075281

[44] Chaudhary N , Weissman D, Whitehead KA. mRNA vaccines for infectious diseases: principles, delivery and clinical translation. Nat Rev Drug Discovery 2021; 20(11):817–38. https://doi.org/10.1038/s41573-021-00283-5 PMC8386155 34433919

[45] Anjorin AA , Odetokun IA, Nyandwi JB et al Public health surveillance for adverse events following COVID-19 vaccination in Africa. Vaccines (Basel) 2022; 10(4):546. https://doi.org/10.3390/vaccines10040546 35455295 PMC9032114

